# *Anopheles* Imd Pathway Factors and Effectors in Infection Intensity-Dependent Anti-*Plasmodium* Action

**DOI:** 10.1371/journal.ppat.1002737

**Published:** 2012-06-07

**Authors:** Lindsey S. Garver, Ana C. Bahia, Suchismita Das, Jayme A. Souza-Neto, Jessica Shiao, Yuemei Dong, George Dimopoulos

**Affiliations:** W. Harry Feinstone Department of Molecular Microbiology and Immunology, Bloomberg School of Public Health, Johns Hopkins University, Baltimore, Maryland, United States of America; Stanford University, United States of America

## Abstract

The *Anopheles gambiae* immune response against *Plasmodium falciparum*, an etiological agent of human malaria, has been identified as a source of potential anti-*Plasmodium* genes and mechanisms to be exploited in efforts to control the malaria transmission cycle. One such mechanism is the Imd pathway, a conserved immune signaling pathway that has potent anti-*P. falciparum* activity. Silencing the expression of *caspar*, a negative regulator of the Imd pathway, or over-expressing *rel2*, an Imd pathway-controlled NFkappaB transcription factor, confers a resistant phenotype on *A. gambiae* mosquitoes that involves an array of immune effector genes. However, unexplored features of this powerful mechanism that may be essential for the implementation of a malaria control strategy still remain. Using RNA interference to singly or dually silence *caspar* and other components of the Imd pathway, we have identified genes participating in the anti-*Plasmodium* signaling module regulated by Caspar, each of which represents a potential target to achieve over-activation of the pathway. We also determined that the Imd pathway is most potent against the parasite's ookinete stage, yet also has reasonable activity against early oocysts and lesser activity against late oocysts. We further demonstrated that *caspar* silencing alone is sufficient to induce a robust anti-*P. falciparum* response even in the relative absence of resident gut microbiota. Finally, we established the relevance of the Imd pathway components and regulated effectors TEP1, APL1, and LRIM1 in parasite infection intensity-dependent defense, thereby shedding light on the relevance of laboratory versus natural infection intensity models. Our results highlight the physiological considerations that are integral to a thoughtful implementation of Imd pathway manipulation in *A. gambiae* as part of an effort to limit the malaria transmission cycle, and they reveal a variety of previously unrecognized nuances in the Imd-directed immune response against *P. falciparum*.

## Author Summary

The immune response of the mosquito vector of *Plasmodium* has proven to possess powerful anti-*Plasmodium* defense capabilities. As the major regulators of these immune responses, signaling pathways, particularly the Imd pathway which seems especially capable of eliminating malaria parasites, have become attractive candidates targets for malaria-control interventions. Although the general anti-parasitic activity of the Imd pathway has been established, the particular components of the pathway involved and the physiological conditions under which the pathway is capable of limiting infection are mostly unknown. Awareness of these major players and conditions is crucial for adapting the Imd pathway into an intervention strategy. We report that while several members of the Imd pathway are critical for such a response, others are dispensable. We also show that timing of the response with regard to infection and intensity of infection exposure both influence the effectiveness of an Imd-derived anti-*Plasmodium* response while the status of the gut flora does not. Taken together, this data lays the essential groundwork for effective intervention based on manipulation of this pathway that can severely limit mosquito infection with human malaria parasites.

## Introduction

Malaria remains one of the world's most devastating infectious diseases, and its successful control will require a multifaceted approach involving a combination of multiple strategies [1], [2]. A multitude of potential methods to prevent mosquito-to-human transmission exist, including several based on manipulating the mosquito's immune response. Despite the lack of an adaptive immune system, mosquitoes are able to quickly and efficiently mount an innate immune response against bacteria, viruses, fungi, and parasites, including *Plasmodium*. In fact, such an immune response is responsible for one of the most dramatic reductions in the parasite population during the *Plasmodium* life-cycle: a log-fold loss in parasite numbers as ookinetes cross the mosquito's midgut cells and develop into oocysts. This drop in parasite number is due, at least in part, to an effective cellular and systemic anti-*Plasmodium* immune assault. Boosting the mosquito's immune response to a level that would fully eliminate this already vulnerable parasite stage could effectively terminate the malaria transmission cycle.

Immune signaling pathways are key regulators of insect immune defenses and are therefore attractive candidates for genetic modification to create a mosquito with an immune response that overwhelms the parasite. Although the Toll and Jak-Stat pathways control immune attacks that limit the development of *P. berghei*, *P. falciparum*, and/or *P.vivax* [3]–[6], the immune deficiency (Imd) signaling pathway has emerged as the most effective pathway in terms of activity against the human malaria parasite. We have previously shown that over-activating the Imd pathway by silencing the gene encoding its negative regulator, Caspar, or over-expressing the gene encoding the REL2 transcription factor confers almost complete protection from cultured *P. falciparum* in laboratory reared *A. gambiae*, *A. stephensi*, and *A. albimanus*; others have observed a requirement for PGRP-LC, one of the receptors activating the Imd pathway, in the response of wild-caught *A. gambiae* to field isolates of *P. falciparum*, and still others have reported a role for REL2, in the response of the recently colonized Ngousso *A. gambiae* strain to NF54 *P. falciparum* [4], [7]–[9]. Thus, the Imd pathway has emerged as a critical factor in the ability of malaria-transmitting mosquitoes to kill *P. falciparum*, and this influence is translatable from laboratory to field, most likely across multiple *Anopheles* species and *Plasmodium* strains.

We have shown that an early activation of the REL2 transcription factor in the midgut tissue, at 6–14 hours after ingestion of an infected blood meal is affective at targeting *P. falciparum* [10]. A snapshot of the mosquito's global gene regulation, potentiated by over-activation of the Imd pathway (via *caspar* silencing), indicated that thioester-containing protein 1 (TEP1), fibrinogen immunolectin 9 (FBN9), and a leucine-rich repeat family member (LRRD7/APL2) are three of the major players in the *Plasmodium* killing that occurs in *caspar*-silenced mosquitoes of the *A. gambiae* Keele strain [4], [10]. In addition, another leucine-rich repeat family member, APL1A, was identified as a REL2-controlled antiplasmodial effector in the Ngousso strain [8]. These genomic and functional studies of downstream effectors mediating the Imd-dependent infection phenotypes have provided important mechanistic insights and have encouraged the hypothesis that vector control methods targeting the Imd pathway are inherently multifaceted at the effector level. However, the high degree of biological complexity that characterizes the vector-parasite interaction requires a deeper understanding of Imd pathway-directed killing of *Plasmodium* at the physiological level.

In this study, we addressed some of the remaining key questions regarding the impact of the Imd pathway on *P. falciparum* infection in the *A. gambiae* Keele strain, with the goal of facilitating the identification of optimal strategies for Imd pathway manipulation.

**Figure 1:**
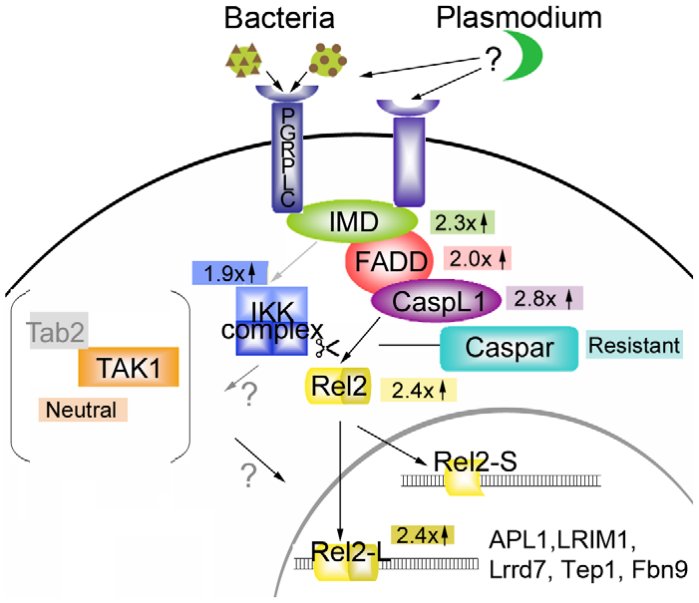
Anopheles Imd pathway model. Components of the Imd pathway explored in this study or others are represented by different colored shapes. Black arrows or lines indicate known interactions or translocations. Gray arrows indicate potential interactions based on *D. melanogaster* studies. The gray bracketed area indicates the molecules possibly involved in other responses, but not the responses against *P. falciparum*. Numbers and arrows within colored blocks indicate the -fold change in *P. falciparum* infection that results when the corresponding pathway member is silenced. The list of genes inside the nucleus portion of the diagram shows those known to be active against *Plasmodium* and whose expression has been shown by our studies to be REL2-regulated.

We first addressed the contribution of specific Imd pathway components in the anti-*Plasmodium* defense. Existing data have implicated the negative regulator Caspar, the transcription factor REL2, and the pattern recognition receptor PGRP-LC in the defense against malaria so we concentrated our efforts on the Imd pathway components known in *Drosophila* to interact with or between these components and that have a clear 1-to-1 ortholog in *Anopheles* (Figure 1), though *Anopheles* immune signaling pathways do not necessarily mimic those in *Drosophila*. As examples, mosquitoes do not have an ortholog of Dif, one of the transcription factors downstream of the Toll pathway in *Drosophila* [11], *Anopheles* possesses two functional isoforms of REL2, while flies have only one [12] and TAB2 does not have a reliable ortholog in *A. gambiae*. We specifically sought to identify the range of Imd pathway genes that have an impact on *P. falciparum* infection, so we assessed the contribution of each potential pathway member to both the natural (infection only) and the artificially enhanced (infection plus *caspar* silencing) immune response against *P. falciparum*.

We then determined which parasite life-cycle stages are vulnerable to an Imd pathway-derived attack. After it enters the midgut lumen, *Plasmodium* completes its fertilization to form a zygote, transitions to the motile ookinete form, penetrates the midgut epithelium, and transforms into the oocyst stage. Since most assessments of immunity enumerate parasites at the oocyst stage, it is often unknown which stage(s) are targeted by anti-*Plasmodium* mechanisms, yet this information could drastically affect how an anti-*Plasmodium* strategy is designed.

It has long been known that mosquitoes' endogenous bacteria can affect *Plasmodium* development [13], [14], but a recent cohort of studies exploring the tripartite interactions between vector, parasite, and the vector's intestinal microflora at the molecular level have revealed complexities that can drastically affect immune responses and *Plasmodium* densities in mosquitoes [7], [9], [15], [16]. An important consideration for any effector mechanism that is acting against both parasites and bacteria (such as the Imd pathway) is that its activity may be elicited either directly by the parasite or indirectly by the proliferation of gut bacteria that occurs concurrently with blood-feeding, and how that elicitation occurs is likely to be crucial for intervention planning. This concern is particularly important for the Imd-driven response to *P. falciparum* because gut-specific over-expression of REL2 in *A. stephensi* has a significant effect on *P. falciparum*, highlighting the immune contribution of tissues harboring bacteria [10]. Therefore, in the present study we also asked whether bacteria are required for the effectiveness of *caspar* silencing in limiting *P. falciparum*.

To date, all analyses of the effect of the Imd pathway and its downstream components on *P. falciparum* infection have utilized an exceptionally virulent strain of *P. falciparum* that is capable of producing high intensities of infection. Recent work from Mendes *et al.* [17] has revealed differential gene expression profiles in mosquitoes that were given higher or lower parasite exposures, suggesting that the anti-*Plasmodium* response can vary with the infection intensity. To assess the potential role the Imd pathway in this differential response, we also assessed the outcome of infection following three different levels of parasite exposure in mosquitoes in which several Imd pathway members and downstream effectors had been silenced.

## Results

### Components of the Imd pathway modify the Caspar-mediated anti-*Plasmodium* defense

To assess the contributions of Imd pathway components to the anti-*Plasmodium* defense, we used an RNA interference approach to silence individual genes in order to determine whether each was necessary for *P. falciparum* infection. A double-silencing assay was also performed to determine whether those components with an effect on *P. falciparum* could reverse the decreased infection that was observed when *caspar* was silenced, thereby identifying any factors that are essential for the *caspar* silencing-mediated immune defense in addition to the routine defense against *P. falciparum*.

**Figure 2:**
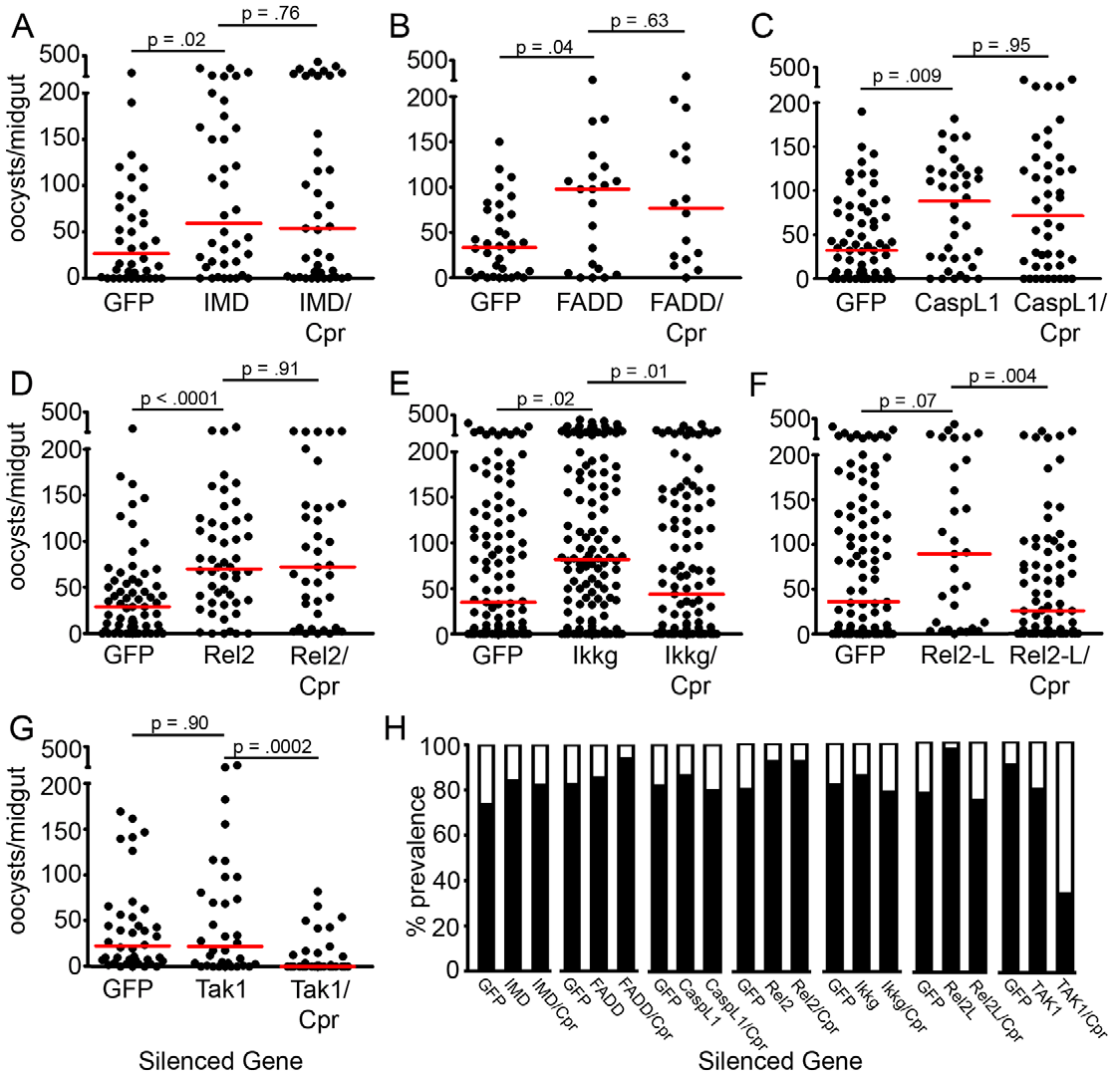
Some members of the Imd pathway have an effect on P. falciparum infection. (A–G) Dots represent individual oocyst counts following the indicated RNAi treatment; horizontal red bars represent the median number of oocysts per gut. P-values were derived from Mann-Whitney statistical tests and appear above each treatment and refer to that treatment as compared to the GFP dsRNA-treated control. Additional statistical analyses appear in Table S1) Filled portion of bars represent the % of all mosquitoes harboring at least one oocyst; open portion represents those in the group that were uninfected. All assays represent two to three independent biological replicate. Cpr, Caspar. (H) Prevalence of *P. falciparum* infection following the indicated RNAi treatment.

**Figure 3:**
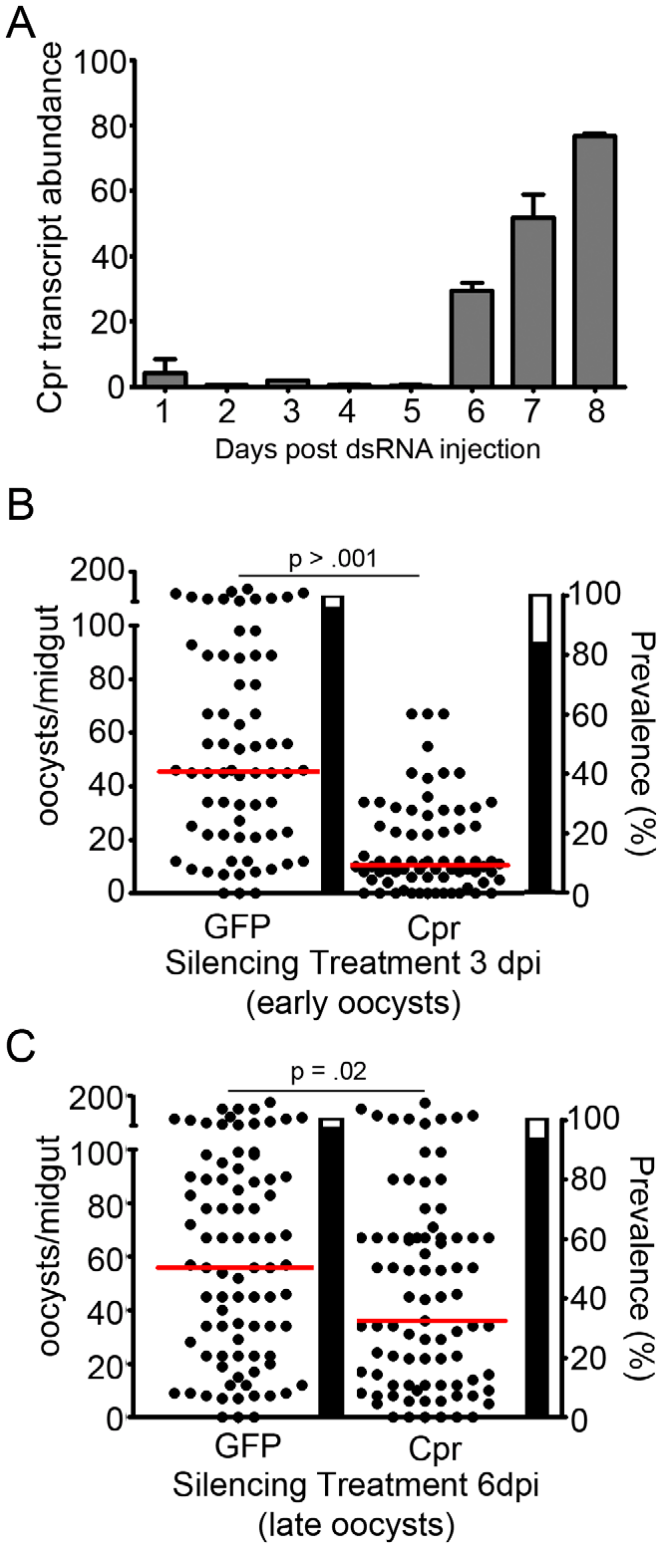
Caspar silencing also influences early and late oocysts. (A) Time course of *caspar*-silencing efficiency, quantified using real-time quantitative PCR. Gray bars represent the % *caspar* expression at each given time point as an average of two replicates, and error bars reflect the standard error between those replicates. Cpr, Caspar. (B) Infection intensity of mosquitoes silenced for *caspar* at 3 days post-infection (dpi). Since ookinetes invade the midgut at 24 hours post-infection this time point targets early oocysts. (C) Infection intensity of mosquitoes silenced for *caspar* at 6 days post-infection. Since sporozoites begin to emerge from the oocyst at 7–8 days post infection, this time point targets late oocysts. For both, dots represent individual oocyst counts following the indicated RNAi treatment; horizontal red bars represent the median number of oocysts per gut. Assays represent two to three independent biological replicates and were subject to Mann-Whitney statistical tests. P-values appear above each treatment and refer to that treatment as compared to the GFP dsRNA-treated control. Additional statistical analyses appear in Table S2. Filled portion of bars represent the % of all mosquitoes harboring at least one oocyst; open portion represents those in the group that were uninfected. Cpr, Caspar.

**Figure 4:**
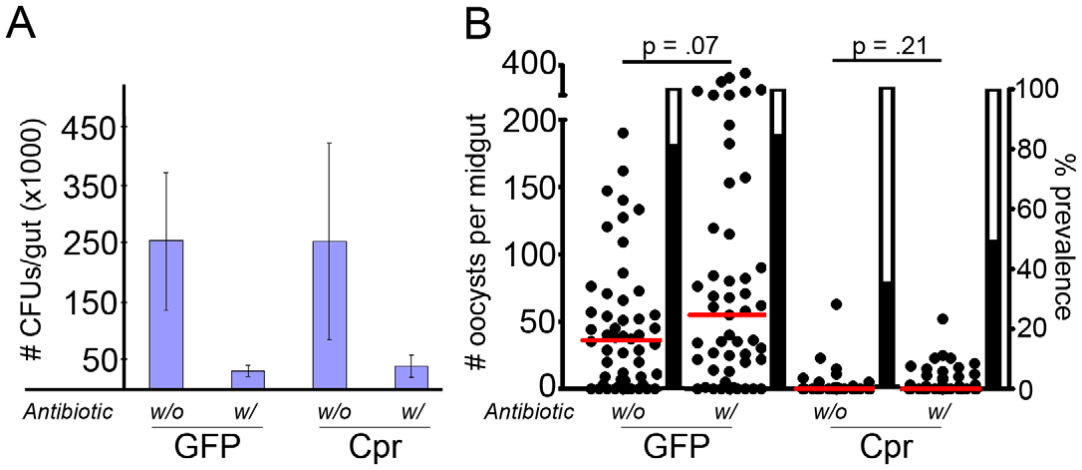
Caspar-mediated killing of P. falciparum is not dependent on midgut bacteria. (A) Blue bars represent bacteria colony-forming unit (CFUs) from midguts of mosquitoes undergoing the indicated treatments. Pluses and minuses indicate the presence or absence of antibiotic in GFP or Cpr dsRNA treated group. Each bar represents the average of at least 15 mosquitoes tested, with each mosquito's CFU count determined by averaging counts from three serial dilutions. Bars represent the standard deviation for all mosquitoes in a given treatment group. Cpr, Caspar. (B) Dots represent individual oocyst counts following the indicated RNAi treatment; horizontal red bars represent the median number of oocysts per gut. Pluses and minuses indicate the presence or absence of antibiotic in the GFP or Cpr dsRNA-treated group. Assays represent three independent biological replicates and were subject to Mann-Whitney statistical tests. P-values appear below each treatment and refer to that treatment as compared to the GFP dsRNA-treated control. Additional statistical analyses appear in Table S3. Filled portion of bars represent the % of all mosquitoes harboring at least one oocyst; open portion represents those in the group that were uninfected. Cpr, Caspar.

Silencing of the Imd pathway factors *imd*, *fadd*, *caspL1* (*dredd*), and *rel2* resulted in median *P. falciparum* oocyst infection intensities that were at least two-fold greater than those of the control group treated with dsRNA against GFP (Figure 2A–2D, Table S1). Of these genes, only silencing of *rel2* had a significant effect on infection prevalence (Figure 2H and Table S1). Co-silencing of the four pathway members (*imd*, *fadd*, *caspL1*, and *rel2*) with *caspar* completely reversed the typical resistance to infection observed when only *caspar* was silenced; i.e., median infection intensities were not significantly different from those of the single-silenced groups and did not exhibit the absence of infection typically observed following *caspar* silencing (Figure 3) and [4].

Single-silencing of *ikk-gamma* increased the median number of oocysts per midgut by 2.3-fold over that of the GFP dsRNA-treated control mosquitoes, yet co-silencing *ikk-gamma* with *caspar* only partially reversed the *caspar*-silencing resistance; silencing of *ikk-gamma* in conjunction with *caspar* returned the median infection intensities to control levels (54% of the single-silencing levels) (Figure 2E). Similarly, silencing *rel2-L* increased the median oocyst load by 2.47-fold and significantly increased prevalence, yet co-silencing did not reverse the effect of *caspar* silencing; median intensities in the *rel2-L*/*caspar*-silenced mosquitoes were only 70% of the control levels (28% of the single-silencing levels) (Figure 2F and 2H).

Depletion of the TAK1 protein had no significant effect on *P. falciparum* infection; median oocyst levels in silenced mosquitoes reached 22 oocysts per mosquito, nearly identical to the median of 22.5 oocysts per mosquito in GFP dsRNA-treated controls (Figure 2G). Accordingly, silencing *caspar* still resulted in significant repression of *P. falciparum* intensity and prevalence, even when *tak1* was silenced (Figure 2G and 2H). TAK1 is a branch point bridging the Imd and JNK pathways; therefore, to ensure that *caspar* is controlling only the Imd pathway, we assessed how the depletion of *jnk* would affect *P. falciparum* and found no effect (data not shown).

### The Imd pathway is primarily active against *P. falciparum* pre-oocyst stages

Although *Plasmodium* parasites are susceptible to mosquito immune responses at multiple stages of their development, previous analyses of *caspar* silencing have measured only the changes in oocyst numbers, an approach that cannot distinguish between activity against a specific stage and that against multiple stages. To discover which stage(s) of development is (are) halted by *caspar* silencing, we developed an assay in which the time of dsRNA injection was varied. In this assay, peak silencing occurred only after most parasites had matured to a specific stage. Time course analysis of *caspar* silencing revealed that efficient depletion of *caspar* mRNA throughout the mosquito body could be achieved within a day of injection, and the effect would persist for at least 6 days (Figure 3A). Based on this kinetic profile, we administered dsRNA against *caspar* or GFP at 3 and 6 days post-infection to specifically target early and late oocysts, respectively. Previous analyses of Caspar have utilized silencing at 3 to 4 days pre-infection, a time at which one can also assess the effect on ookinetes (Figure 2) [4]. We have also previously shown that over-expression of REL2 in the midgut tissue following a blood meal results in the inhibition of pre-ookinete stages and/or killing of ookinetes in the lumen prior to invasion [10]. We cannot, however, exclude the possibility that *caspar*, which is upstream of REL2, also controls a REL2-independent branch of the Imd pathway.

Administered 3 days post-infection, *caspar* silencing significantly reduced the number of developed oocysts when compared to GFP dsRNA-treated controls. Although the mosquitoes were not rendered resistant to infection, prevalence of infection was reduced by 12.3% and the intensity of infection was still severely reduced: GFP-dsRNA treated mosquitoes harbored a median 45.5 oocysts per gut, whereas *caspar*-silenced mosquitoes harbored only 10.5, a decrease of 77.3% (Figure 3B, Table S2A). For comparison, silencing genes encoding negative regulators of the Toll (Cactus) and Stat (Pias) pathways, two pathways involved in the killing of parasites [4], [5], also had an effect on early oocyst development; *caspar* silencing exhibited the strongest degree of infection inhibition, and *cactus* silencing the weakest (data not shown).

In contrast to treatments affecting the pre- and early oocyst stages, silencing treatments designed to target the late-oocyst stages (silencing at 6 days post-infection) were far less effective in limiting the number of parasites per midgut in *caspar*-silenced and control mosquitoes, although, surprisingly, an appreciable degree of infection inhibition was still observed. In these experiments, the median number of surviving oocysts in the *caspar*-silenced mosquitoes was 36, as compared to 56 in the GFP dsRNA-treated mosquitoes, representing a decrease of 45.7% while prevalence was not impacted (Figure 3C, Table S2B).

### The Caspar silencing-mediated anti-*P. falciparum* defense is independent of the midgut microbiota

Several studies have illuminated the importance of tripartite interactions between the mosquito's immune system, the parasite, and the mosquito's intestinal flora during *Plasmodium* infection [7], [9], [15], [16]. For some anti-parasite gene regulation and killing mechanisms, the presence and appropriate composition of the bacterial populations in the gut are required [9], [15], [16]. In *Drosophila*, the Imd pathway is a primary regulator of the response against intestinal bacteria [18]–[20], and we have observed that REL2 over-expression exclusively in the mosquito midgut offers resistance to parasites [10], leading us to question whether bacteria are necessary for the activation of the Imd pathway during *caspar* silencing, or whether the Imd pathway activation provided by the bacteria under normal conditions is simply supplanted by the artificial Imd pathway activation mediated by *caspar* silencing. To answer this question, we administered an antibiotic cocktail to mosquitoes before treating them with dsRNA against *caspar* or GFP and subjecting them to *P. falciparum* infection. We have previously shown that antibiotics can be used to eliminate the majority of the bacterial population from the mosquito midgut [9], [21]. As had previously been shown, we found that GFP dsRNA-treated mosquitoes were more susceptible to *Plasmodium* infection if they had been pre-treated with antibiotics; aseptic mosquitoes harbored almost twice as many oocysts as their GFP dsRNA-treated septic counterparts (Figure 4A, Table S3). However, the lack of bacteria in antibiotic-treated mosquitoes had no effect on the ability of *caspar* silencing to severely limit parasite development; both septic and aseptic mosquitoes treated with dsRNA against *caspar* displayed median oocyst levels of 0 and had much lower prevalence of infection than did either GFP dsRNA-treated group, though not significantly different from one another (Figure 4B, Table S3).

### The efficiency of the pro-and anti-parasitic responses obtained by silencing Imd pathway components and downstream effectors is dependent on infection intensity

**Figure 5:**
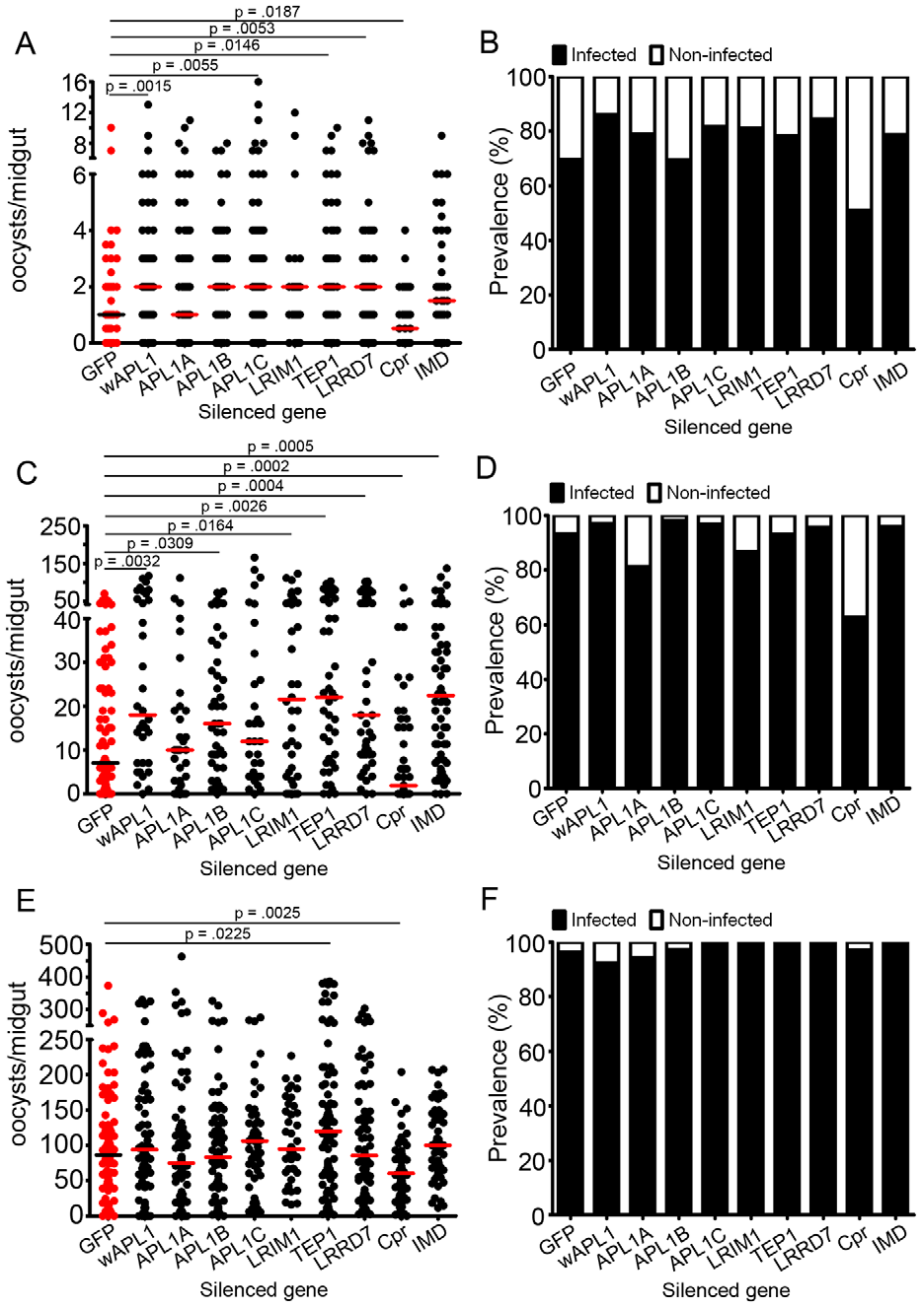
Imd pathway components and effectors differ in their ability to affect P. falciparum infections of high, medium, and low infection intensities. Intensity of *P. falciparum* oocysts in *A. gambiae* silenced for given genes and subjected to low (A), medium (C) or high (E) infection exposures. Bars represent median numbers of oocysts per midgut, and dots represent individual midgut oocyst counts. Assays represent at least three independent biological replicates and were subject to Mann-Whitney statistical tests. P-values appear above each treatment and refer to that treatment as compared to the GFP dsRNA-treated control. Non-significant p-values were not included in the figure. Additional statistical analyses appear in Table S4. D-F: Prevalence of infection in *A. gambiae* subjected to low (B), medium (D) and high (F) loads of *P. falciparum*. Filled portion of bars represent the % of all mosquitoes harboring at least one oocyst; open portion represents those in the group that were uninfected. wAPL1 (whole APL1) dsRNA – dsRNA for a conserved region of APL1 genes, which results in the silencing of all three APL1 proteins (APL1A, APL1B and APL1C).

Earlier studies have featured a model system composed of a highly virulent parasite strain and highly susceptible mosquito strain. While we have previously shown that the Imd pathway is effective against *P. falciparum* in other *Anopheles* strains [4], recent data have revealed the importance of infection intensity in the generation of immune responses directed against *Plasmodium* [17]. To determine whether Imd-derived responses are more or less effective against different levels of infection, we subjected mosquitoes to a standard administration of dsRNA against various Imd pathway members and downstream effectors; then, at 3 to 4 days after the dsRNA injection, we fed them on *P. falciparum*-infected blood with a more dilute or more concentrated gametocyte culture than that used in the standard protocol. Oocyst counts indicated that, indeed, low-, medium-, and high-intensity infections had been achieved, with roughly log-fold differences (median = 1, 7, and 86, respectively) from the levels in GFP dsRNA- treated control mosquitoes (Figure 5, Tables S4A–C) and that the infection phenotypes observed following silencing were not consistent for the three levels of infection intensity (Figure 5). For example, silencing all forms of *apl1* significantly increased the infection levels (when compared to the GFP dsRNA-treated group) only at low (a 2-fold increase) and medium (a 2.6 -fold increase) infection intensities. Specific paralog silencing suggested that APL1C was most effective in influencing low-level infections (a 2-fold increase), while APL1B was the most influential paralog at medium infection intensities (a 2.3-fold increase). In our experiments, APL1A depletion had no effect on *P. falciparum* infection under any condition.

As was seen when we silenced all forms of *apl1*, knockdown of another member of the leucine-rich repeat-containing family, *lrrd7*, was effective in low- and medium-, but not high-intensity infections. Importantly, we noted that both the prevalence and intensity of infection were significantly increased by silencing *lrrd7* prior to low levels of exposure to *P. falciparum*, but only infection intensity was significantly altered by *lrrd7* silencing prior to medium-level exposure. Thus, the contribution of a gene to the anti-*Plasmodium* response is not only dependent on the level of exposure but also on the method of data collection used (Figure 5 and Tables S4A–C).

As other studies have shown, we found no discernible effect of a third LRR-containing effector, LRIM1, during *P. falciparum* infection at either low or high intensity. However, we surprisingly observed that in the context of a medium-level infection, silencing of LRIM1 did substantially enhance infection (a 3.1-fold increase) (Figure 5).

Interestingly, silencing the major pathway component, Imd, was only effective at increasing infection intensities at high infection levels (a 3.2-fold increase), the level closest to that at which pathway analysis is typically performed (Figure 5). Nevertheless, *caspar* silencing was effective in limiting infection, regardless of the exposure dose; in essence, *caspar* silencing was effective in limiting low-, medium- and high-intensity *P. falciparum* infections. The only other tested effector to display this consistent effect was TEP1 (Figure 5).

As a control, we checked the efficiency of the gene silencing of the Imd pathway components and downstream effectors genes by measuring the expression of their gene-specific RNAs and observed a reduction of 48–92% in their regular expression patterns (Table S5).

## Discussion

Despite the fact that the negative regulator of the Imd pathway, Caspar, controls a branch of the mosquito immune system that determines the mosquito's resistance to human malaria parasites, until now little has been known about the genes/proteins controlled by this regulator, when they exert their effects on the parasite, and whether this activity is dependent on a tripartite relationship with endogenous bacteria or is influenced by infection intensity. The answers that we have obtained to these essential questions about the biology of Caspar should facilitate the use of the Imd pathway for the development of malaria control strategies.

### The Imd pathway contains an anti-*P. falciparum* module

In the present study, we first addressed the contribution of selected components of the Imd pathway to the normal mosquito response against *P. falciparum* (single-silenced and infected) and the Caspar-controlled response against *P. falciparum* in relation to other Imd pathway factors (double-silenced and infected). These experiments revealed three different phenotypes for Imd pathway members: 1) full participation in both anti-*P. falciparum* responses and *caspar*-mediated resistance; 2) weaker participation in anti-*P. falciparum* responses, with unclear contribution to the response directed by *caspar*; and 3) no participation in anti-*P. falciparum* immunity.

For the first group of components, silencing genes encoding four of the known components of the Imd pathway (Imd, FADD, CaspL1 and REL2) resulted in a significant increase in the intensity of the midgut infection when compared to GFP dsRNA-treated controls and an ability to reverse the resistance caused by *caspar* silencing. Importantly, co-silencing Imd, FADD, CaspL1, and REL2 with *caspar* gave median infection intensities that were almost identical to those of the single-silenced groups. This result suggests a complete dependence of the *caspar*-silenced infection phenotype on these pathway members. This finding is not surprising, since *Drosophila* Caspar has been suggested to regulate the Dredd-dependent cleavage of REL2, and Imd and FADD are co-activators of Dredd [22]. Thus, the four components that produced both the strongest single-silencing phenotype and the most complete reversion in double-silencing experiments were also the components that have been hypothesized to interact most directly with Caspar.

In contrast, the second group of components produced reasonable increases in infection level in response to single silencing, yet only a partial reversion of the *caspar* phenotype. Reduced levels of IKK-gamma led to an appreciable increase in oocyst load when compared to control silencing treatments; the co-silencing of *caspar* and IKK-gamma showed a mild reversion of the *caspar* phenotype, but to levels that were not significantly elevated over those for the GFP dsRNA-treated control (as was consistently observed for the four genes in the first group). This result could suggest either a lesser involvement in *caspar*-dependent immune responses than for the other components, a dual role for IKK-gamma in *caspar*-dependent and *caspar*-independent responses (the independent form interfering with a complete reversion of the dependent), or a *caspar*-independent role in antagonizing *P. falciparum* response that overwhelms the agonistic output of Caspar.

REL2-L is also a member of the second group. Experiments in the *Drosophila* model system have strongly suggested that Imd pathway activation results in the cleavage of the ankyrin repeat region of the C-terminus of Relish, likely by Dredd, freeing the active form of Relish for translocation to the nucleus [23], [24]. In *Anopheles*, both a full form of the Relish ortholog (REL2-L) and a short form lacking ankyrin repeats (REL2-S) are independently transcribed [12]. Because there are no unique sequences for targeting only REL2-S with dsRNA, the distinct roles of the two forms are unclear. However, because the long form can be targeted independently of the short form (by targeting dsRNA to the region encoding the ankyrin repeats) the contributions of REL2-L to a phenotype can be quantified independently of the contributions of all REL2 forms. Mitri and colleagues [8] have reported that silencing only *rel2-L* has no effect on the prevalence of *P. falciparum* infection, although infection intensities were not reported in their study. We observed in the present study that silencing *rel2-L* had a noticeable effect on oocyst burden in single-silencing assays but did not result in the same potent immune response against *P. falciparum* as did silencing all forms of *rel2*. In addition, while co-silencing of *caspar* and *rel2-L* did not result in complete refractoriness, double-silencing was unable to reverse the phenotype, even to the level of infection intensity observed for the GFP dsRNA-treated control group, much less to the single-silencing levels observed when all forms of *rel2* were targeted (Figure 1D). The inability to target REL2-S leaves the identification of distinct roles for the short and long forms still unclear. We can therefore only conclude that both forms of REL2 are able to participate in the normal response of *A. gambiae* to *P. falciparum*, with the short form being a major constituent of *caspar*-mediated resistance to that parasite and the long form a more minor component.

The third group of components showed no effect on infection when either single-silenced or double-silenced and had no effect on the outcome of *caspar* silencing. In *Drosophila*, TAK1 is a kinase that is able to activate both JNK and IKKg [25], [26]. Our data showed no observable role for TAK1 in the defense against *P. falciparum* and no requirement for TAK1 in the *caspar*-silencing infection phenotype. Caspar is purported to control the cleavage of REL2 by CASPL1/Dredd [22]; TAK1's role is more likely to be that of an activator/mediator of the branch point between the Imd and JNK pathways [27]–[29]. Therefore, TAK1 may be dispensable during the activation of REL2 that occurs in *caspar*-silenced mosquitoes and, if so, it should not be considered a major target in future applications. Consistent with this conclusion that TAK1 is not a major player in anti-*P. falciparum* responses, our further investigation into the JNK pathway side of TAK1's responsibilities revealed that *P. falciparum* is unaffected by JNK silencing. Since this screen is directed toward anti-*Plasmodium* responses, we cannot remark on the contributions of TAK1 (or other Imd pathway genes) during other infections in *Anopheles*.

Taken together, these data suggest that the proteins hypothesized to act most intimately with Caspar (Imd, FADD, CASPL1, and REL2) are the most effective players in the mosquito defense against *P. falciparum* and that the Imd pathway is uniquely potent against this parasite. Based on these data, we propose a model of the *A. gambiae* Imd pathway (illustrated in Figure 1) in the context of *P. falciparum* infection. Since this model was generated on the basis of data from RNAi-based gene silencing, researchers must bear in mind the caveats associated with this methodology: i.e., the extent of the silencing is dependent on the silencing efficiency, mRNA turnover, tissue specificity of expression, and the ability of the dsRNAs to reach the appropriate tissues. It is also possible that additional components not included in this targeted screen are part of the anti-*Plasmodium* Imd signaling module; including known Imd pathway genes not assessed in this screen, unknown genes serving as novel members or unknown genes serving as a functional equivalents of known genes (such as TAK1) in mosquitoes. Nevertheless, the strong infection-intensity phenotypes obtained by silencing multiple Imd pathway members suggest that this module within the pathway is a major player in the anti-*P. falciparum* defense employed by *A. gambiae*.

### The Imd pathway is primarily active against pre-oocyst *P. falciparum* stages

Previous analyses of Caspar and Cactus have utilized a standard silencing protocol developed to assess immune responses directed against the ookinete stage of *Plasmodium* [3], [4]. This protocol is widely used because the ookinete stage represents a bottleneck in terms of parasite population that is thought to be caused at least in part by the mosquito's immune responses. However, depending on the silencing kinetics of a particular gene, this approach either ignores immune molecules that are effective at later stages, when transcript levels of the target gene have recovered, or is unable to distinguish effects at earlier or later stages because silencing occurs throughout. These complications were made apparent by a study that found a role for the Stat pathway against early oocysts but not ookinetes [5]. Because the silencing of *caspar* occurs quickly, but mRNA levels recover within 5 to 6 days (Figure 3A), identifying the parasite stages affected by the Imd pathway can be achieved by varying the times at which dsRNA is injected. This method has revealed that ookinetes are most affected by Imd pathway activation, resulting in mosquito resistance to the parasites, but there is also a significant contribution of the pathway to limiting early oocysts (Figure 3B) [4]. In addition, *caspar* silencing is weakly, but significantly, effective in limiting the development of late oocysts, in agreement with our previous finding that REL2 activation in the midgut tissue also results in the inhibition of late oocysts, and possibly sporozoite stages, of *P. falciparum* [10].

Together, these studies indicate that there is an extended window of opportunity in the parasite lifecycle for the mosquito to respond to and combat *P. falciparum* by means of the Imd pathway, but there is an optimal time of response within that window. We hypothesize that this timing is the reason that pre-arming the mosquito with downstream effectors by artificially activating the Imd pathway through *caspar* silencing allows the mosquito to mount a rapid, strong immune response in the midgut epithelium and hemolymph that is effective in killing the most susceptible stages of *Plasmodium*. Under normal conditions, the mosquito must first sense infection then activate the Imd pathway, at which point the effectors can respond; the efficiency of killing is reduced as the time required to complete these steps increases. By silencing *caspar*, we can circumvent the detection and activation steps, so the effector mechanisms are quickly established and at the ready for early killing, at the most susceptible stage of the parasite. This hypothesis has been corroborated by our previous study in which we found that an earlier-than-normal enrichment of the REL2 protein through transgenic over-expression resulted in a profound decrease in *P. falciparum* infection [10].

Understanding the importance of timing is clearly crucial for manipulating this biology in future studies addressing the Imd pathway as part of an integrated malaria control strategy. The message from these experiments is that early Imd pathway activity is preferable, and the pre-ookinete stages are the most vulnerable to an attack in the midgut tissue [10]. However, such a response can still be antagonistic to the parasite at later time points in the lifecycle.

### The effect of caspar silencing on *P. falciparum* is independent of gut microbiota

Several studies have reported a significant contribution of the endogenous flora to the generation of a mosquito's anti-parasitic responses. In addition to the direct interaction between bacteria and parasites, the exponential increase in midgut bacterial loads during blood-feeding elicits immune gene expression and activates immune mechanisms at the same time that *Plasmodium* parasites are present in the lumen and invading the midgut epithelial cells [21]. These immune mechanisms attack or limit *Plasmodium* indirectly but adequately [9], [10]. If the resistance to *P. falciparum* is not solely due to immune activation operating via manipulation of Imd pathway expression but is instead dependent on normal activation of the Imd pathway by bacterial PAMPs (that is merely augmented by *caspar* silencing), any control strategy based on the Imd pathway will be affected by the mosquito's individual flora. Detectable infection levels in mosquitoes lacking endogenous bacteria suggest that the lack of negative regulation in the Imd pathway is sufficient to confer resistance to *P. falciparum*. Standard antibiotic treatments eliminate 99% of the endogenous bacteria [8], [9], but it is possible that PAMPs from dead bacteria or shed peptidoglycan still exist in antibiotic-treated mosquitoes. Thus, it is possible that a basal level of bacteria or bacterial components is required to begin Imd pathway activation, and the lack of negative regulation perpetuates the up-regulation of anti-microbial effectors that kill *Plasmodium*. However, the observation that the required basal level of elicitation can occur in mosquitoes with <1% of the normal flora suggests that the differences in bacterial loads or flora composition would have only minimal effect on *P. falciparum resistance* directed by the Imd pathway.

### The anti-*Plasmodium* activity of individual Imd pathway components is influenced by infection intensity

By manipulating the dosage of *P. falciparum* gametocytes available during blood feeding, we have been able to control the average infection intensity in a given group of mosquitoes. These experiments showed that the requirement of a given pathway component or effector, assessed by RNAi, is often dependent on that average infection intensity (but not always, as evidenced by Caspar and Tep1) (Figure 5). Effectors that are known to be required at higher *Plasmodium* doses yet seem ineffective at low doses may not be up-regulated or otherwise activated in control mosquitoes at lower doses. This hypothesis is supported by recent transcriptomic data suggesting that, for some immune genes, expression is dependent on the infection intensity [17]. Alternatively, it is possible that RNAi-based assays are not sufficiently capable of depleting specific transcripts to have an impact on a low-intensity infection; this situation would be particularly true for genes exhibiting a rapid rate of transcription and/or encoding proteins that have potent anti-*Plasmodium* effects at low concentrations.

Effectors known to be required at lower but not higher doses could be influenced by a saturation effect, in which Imd pathway effectors are being produced in quantities that are sufficient to kill lower numbers of parasites but insufficient to deal with infections above a certain level. This would be the case if only a small, finite number of anti-*Plasmodium* effectors are produced and therefore available for combating parasites. This type of effector would not have an effect on infections at high intensities in control mosquitoes, and therefore silencing the gene(s) that encode them would show no effect. Inefficiency of some effectors at higher *Plasmodium* doses may also be governed by infection-intensity gene regulation if high levels of infection cause those genes to be down-regulated, as in, for example, a feedback loop. Such genes would theoretically be produced at sufficient levels in control mosquitoes during low- or medium-level infections but would be down-regulated, and therefore less effective, at higher doses.

Our data suggest that the medium- intensity infection levels are optimal for studying anti-*Plasmodium* defenses under laboratory conditions, since measurable effects can be seen for most genes, and statistical analyses can be made (in contrast to very low-infection assays). However, our data also indicate that looking only at medium-level intensities can lead to unfounded assumptions about the physiological relevance of the examined genes during infection under natural field conditions, and complexities of pathway regulation can be masked.

Our study also offers a number of interesting and novel insights into other aspects of anti-*Plasmodium* defenses that have not been addressed in previous studies using only standard infection intensities:

First, in our assessment of the requirement for LRIM1, LRRD7, and all three paralogs of APL1, we found that some components were necessary for low-, medium- or high-level infection exposures, but none were required at all levels of exposure, perhaps reflecting redundancy or shared roles among this group (Figure 5). How those roles are assigned or regulated with respect to the number of successful ookinete invasion events has yet to be determined. Our analysis of the LRR-containing proteins revealed that the APL1 paralogous genes behaved differently in our experiments than in those reported by Mitri et al. [8]. While we confirmed their report of a role for APL1 genes in limiting *P. falciparum*, our data are not in agreement with regard to the specific paralogs involved. Most strikingly, we did not find a significant role for the APL1A paralog in the anti-*P. falciparum* immune response (Figure 5), but we did for APL1B or APL1C, depending on the intensity of infection. The APL1 gene family has exhibited a complex sequence evolution, including an exceptionally high degree of polymorphism in some strains, with a recent selective sweep occurring in others [30]. Therefore, although our study confirms a role for APL1 gene family members during *P. falciparum* infection, the differences we saw in regard to which family members are playing the effector role may be explained by the possibility that we are assaying mosquitoes with different versions of APL1 sequences, or the fact that all our infection assays used a significantly higher infection intensity than did those of Mitri et al.

Second, the results of infection exposure have revealed a novel role for LRIM1 during *P. falciparum* infection. LRIM1 was originally identified as a powerful mediator of parasite killing in a rodent malaria model but later shown to have little observable effect on *P. falciparum* [31], [32]. Our data indicate that LRIM1 does contribute to the anti-*P. falciparum* response, but only at medium levels of infection intensity (with a trending but non-significant effect at low intensity) (Figure 5). We believe that the discrepancy between our studies can be explained by the fact that the study showing a lack of contribution of LRIM1 [32] was performed with a different mosquito species and a field *P.falciparum* infection model that generated a low infection intensity (indeed, in our experiments, LRIM1 did not display anti-*Plasmodium* activity at low infection intensity). The fact that LRIM1 has been linked to the TEP1/APL1 anti-*Plasmodium* mechanism [33], [34] suggests that it is very likely to play a role in anti-*Plasmodium* activity, but it is possible that it may not be required (or is redundant) at very low infection intensities, or that RNAi-mediated depletion is not be sufficient to reduce it to a level that would have any impact on a low-intensity infection.

Third, the fact that *caspar* silencing had an effect at very high infection intensity whereas Imd silencing did not may suggest that other branches of the Imd pathway act upstream of Caspar and in parallel with the Imd receptor. The existence of multiple downstream signal circuits would also explain the robustness of the *caspar* silencing phenotype and the variety of downstream effectors it regulates. However, the identities of such circuits are unknown.

Fourth, we found that no matter how concentrated or dilute a *P. falciparum*-laden blood meal, silencing *caspar* still greatly reduced the resulting oocyst infection and, conversely, silencing *tep1* significantly increased the infection. These results suggest that these two components are essential factors in the anti-*Plasmodium* defense for which no substitutes are allowed, whereas the other factors may be to some degree redundant.

Taken together, our finding suggest that members of the Imd pathway are reasonable first targets for vector-based malaria control interventions, and analyses of such interventions should necessarily include facets of mosquito-parasite infection biology such as the level of the infection and the parasite stage(s) affected. Clearly, some pathway members would be more effective than others: Specifically, Imd, CaspL1, or REL2 would be good positive regulators to manipulate, along with Caspar as the negative regulator, and we have confirmed this conclusion for REL2 using transgenic methodology [10]. The target should be affected (a positive regulator enhanced or negative regulator repressed) within the first 24 after feeding to have the greatest effect on the ookinete stage, and the treatment could be more effective if it were designed to prolong the enhancement/repression through to the pre-oocyst stage. The fact that the mature oocyst stage was marginally affected by the Imd pathway means that an enhancement/repression lasting longer than 3 to 4 days post-feeding may be unnecessary but beneficial; this timing would be amenable to adjustment in order to compensate for a low impact on mosquito fitness. In a laboratory setting, transient activation of the Imd pathway (via RNAi or *caspar* and transgenic over-expression of REL2) has no observable impact on mosquito fitness, yet is potently anti-parasitic [4], [10]. A similar observation has previously been made in the fruitfly *Drosophila* [35]. Moreover, an Imd pathway-based intervention could also be successful, regardless of the status of the endogenous bacteria; such an approach would avoid potential species- or environment-specific pitfalls, and yet the target of the intervention would be able to effectively kill the parasite even at low levels of infection.

Components of the immune response of the mosquito vector of *Plasmodium* have emerged as favorable candidates for targeted malaria-control interventions. However, the immense technical effort required to build such interventions is not trivial, and therefore the preliminary refinement of candidate selection must be thorough. Here we present data that not only identify the biological mediators of the Imd pathway-driven immune response that can render mosquitoes almost completely refractory to *P. falciparum* but also answer major questions about when and how that response is generated. We show that while several members of the Imd pathway are critical for such a response, others are indispensable, and the anti-Plasmodi*um* response decreases in potency as the parasite matures, with the pre-oocyst stages being most vulnerable and the mature oocyst stage the least. We also show that *caspar*-mediated refractoriness can occur without regard to microbial flora or the intensity of the *Plasmodium* exposure, revealing the delicate yet robust control this negative regulator exerts over the Imd pathway and indicating the pathway's potential for broad applicability.

## Materials and Methods

### Ethics statement

This study was carried out in strict accordance with the recommendations in the Guide for the Care and Use of Laboratory Animals of the National Institutes of Health. The protocol was approved by the Animal Care and Use Committee of the Johns Hopkins University (Permit Number: M006H300). Commercial anonymous human blood was obtained from Interstate Bloodbank and used for parasite cultures and mosquito feeding and informed consent was therefore not applicable. The Johns Hopkins School of Public Health Ethics Committee has approved this protocol.

### Mosquito rearing

Keele strain *A. gambiae* mosquito larvae were raised in 30×34 cm trays (∼250 larvae per tray) with cat food pellets added daily and ground fish food supplemented upon water change. Adults were reared in a 20×20×20 cm^3^ cage and sustained using a 10% sugar solution at 27°C and 80% humidity with a 12-h light/dark cycle according to standard procedures [36].

### RNAi gene silencing

Assays were performed according to a standard protocol [37]. Genes encoding previously identified genes of the *Anopheles* Imd pathway response and genes encoding 1-to-1 orthologs of Imd components known in *Drosphila* were targeted by synthesizing sense and antisense RNAs from ∼300- to 600-bp PCR-amplified gene fragments using the T7 Megascript kit (Ambion) and primers indicated in Table S5. About 69 nl dsRNA (2–3 µg/µl) in water was introduced into the thorax of cold-anesthetized 2- to 4-day-old female mosquitoes using a nano-injector (Nanoject, Drummond) with glass capillary needles according to Blandin et al. [38]. Double-silencing experiments were performed in the same manner, except that dsRNAs targeting each gene were mixed and concentrated so that each dsRNA was present at 2–3 µg/µl. Depletion of dsRNA targets after silencing was quantified; silencing efficiencies are given in Table S5.

### Infection with Plasmodium

Mosquitoes were fed on NF54 gametocytes in human blood through a membrane feeder at 37°C at various time points, depending on the stage being examined: 3–4 days after dsRNA injection for assessment of ookinetes (Figures 2, 4, and 5), 2 days prior to dsRNA injection for early oocysts (Figure 3B), and 6 days prior to dsRNA injection for late oocysts (Figure 3C), and at various dilutions for high-, medium- and low-infection exposures. All mosquitoes were subsequently maintained at 24°C until 7 to 8 days post-feeding, when midguts were dissected and stained with 0.1% mercurochrome in PBS, and oocyst numbers were recorded using light microscopy (Olympus). Each assay was done with at least 25 mosquitoes, and data represent the results of at least three independent assays. P-values were determined using Mann-Whitney tests; further statistical analysis was performed using Fisher's Exact test and Kruskal-Wallis tests with Dunn's Multiple Comparison Summary (Tables S1, S2, S3, S4).

### Time course analysis and real-time quantitative PCR expression analysis

Mosquitoes were silenced for *caspar* or GFP (as a control) 2 days after emergence and incubated under normal conditions. Each day at the same time, ∼10 mosquitoes from each group were collected and homogenized for total RNA extraction. Assays were then performed according to a standard protocol [37]. Total RNA from adult females was extracted using the RNeasy kit (QIAGEN), quantified using a Beckman DU640 spectrophotometer, and subjected to reverse transcription using Superscript III (Invitrogen) with random hexamers. Real-time quantification was performed using the QuantiTect SYBR Green PCR Kit (Qiagen) and ABI Detection System ABI Prism 7000. Primer sequences are given in Table S5. All qPCR reactions were performed in triplicate; to verify the specificity of the PCR reactions, melting curves were obtained for each data point. The levels of expression in gene-silenced samples were determined by normalizing the cDNA levels using the ribosomal protein S7 gene and compared to controls treated with dsRNA against GFP.

### Antibiotic treatment assays

Groups of 80–100 recently emerged (>1-day-old) female *A. gambiae* mosquitoes were sequestered in pint-capacity paper cups and maintained on either 10% sucrose or 10% sucrose supplemented with 10 µg/mL penicillin-streptomycin and 15 µg/mL gentamicin [9]. For each replicate, 5–10 midguts from each group were dissected and homogenized in PBS, and serial dilutions were plated on LB agar to confirm a 1,000-fold reduction in bacterial loads when antibiotics were administered in the sucrose solution. Comparisons of antibiotic-treated and untreated mosquitoes utilized the *Plasmodium* infection, RNA extraction, PCR amplification, and other methods described above. Primer sequences are given in Table S5.

## Supporting Information

Table S1 **Imd pathway phenotypic dissection.**
(DOCX)

Table S2 **(A) Early oocysts. (B) Late oocysts.**
(DOCX)

Table S3 **Caspar silencing with and without antibiotics.**
(DOCX)

Table S4 (**A**) **Effect of silencing of IMD pathway members and effectors on low exposure infections.** (**B**) Effect of silencing of IMD pathway members and effectors on medium exposure infections. (**C**) Effect of silencing of IMD pathway members and effectors on high exposure infections.
(DOCX)

Table S5 **Primers used for dsRNA synthesis and for verification of knockdown of target genes.**
(DOCX)
