# Supplementary material for: Anopheles Imd Pathway Factors and Effectors in Infection Intensity-Dependent Anti-Plasmodium Action
Source: PLoS Pathog. 2012 Jun 7;8(6):e1002737. doi: 10.1371/journal.ppat.1002737 (PMC3369948; doi:10.1371/journal.ppat.1002737)
Supplement: Table S1 — Imd pathway phenotypic dissection. (DOCX) [file ppat.1002737.s001.docx]

**Table S1:** IMD pathway phenotypic dissection

| **Fig. 2**  **IMD pathway oocysts** | **GFP** | **IMD** | **IMD/Cpr** | **GFP** | **CaspL1** | **CaspL1/Cpr** | **GFP** | **Tak1** | **Tak1/Cpr** | **GFP** | **IKKg** | **IKKg/Cpr** |
| --- | --- | --- | --- | --- | --- | --- | --- | --- | --- | --- | --- | --- |
| **n** | 38 | 38 | 39 | 65 | 36 | 44 | 40 | 34 | 38 | 104 | 139 | 107 |
| **Range** | 0-252 | 0-320 | 0-415 | 0-190 | 0-182 | 0-317 | 0-170 | 0-231 | 0-82 | 0-377 | 0-428 | 0- 366 |
| **Prevalence** | 73.7% | 84.2% | 82.1% | 81.5% | 86.1% | 79.5% | 90.0% | 79.4% | 34.2% | 81.7% | 85.7% | 78.5% |
| Fisher’s test p-value | - | 0.118 | 0.232 | - | 0.563 | 0.857 | - | **0.049** | **<0.0001** | - | 0.563 | 0.722 |
| **Median with zeros** | 26.5 | 59 | 54 | 32 | 88 | 71.5 | 22.5 | 22 | 0 | 43 | 82 | 44 |
| % decreased oocysts load | - | - | - | - | - | - | - | 2.3% | 100% | - | - | - |
| % increased oocysts load | - | 122.6% | 103.8% | - | 175% | 123.4% | - | - | - | - | 90.7% | 4.8% |
| Kruskal-Wallis Comparison Summary | ns | ns | ns | * | * | * | *** | *** | *** | * | * | * |
| Dunn’s Multiple Comparison Summary | - | ns | ns | - | p<0.05 | ns | - | ns | p<0.05 | - | ns | ns |
| Mann-Whitney test p-value | - | **0.029** | 0.115 | - | **0.009** | **0.019** | **-** | 0.901 | **<0.0001** | **-** | **0.020** | 0.849 |
| **Median without zeros** | 51 | 104.5 | 85.5 | 41 | 105 | 98 | 30 | 34 | 17^ | 82 | 104 | 72 |
| Kruskal-Wallis Comparison Summary | ns | ns | ns | *** | *** | *** | ns | ns | ns | * | * | * |
| Dunn’s Multiple Comparison Summary | - | ns | ns | - | p<0.05 | p<0.05 | - | ns | ns | - | ns | ns |
| Mann-Whitney test p-value | - | **0.051** | 0.173 | **-** | **0.004** | **0.0005** | **-** | 0.409 | 0.414 | **-** | **0.019** | 0.766 |

| **Fig. 2 cont’d**  **IMD pathway oocysts** | **GFP** | | **Rel2** | **Rel2/Cpr** | **GFP** | **Rel2L** | **Rel2L/Cpr** | **GFP** | **FADD** | | **FADD/Cpr** |
| --- | --- | --- | --- | --- | --- | --- | --- | --- | --- | --- | --- |
| **n** | 64 | | 50 | 37 | 99 | 31 | 79 | 34 | 21 | | 16 |
| **Range** | 0-252 | | 0-280 | 0-222 | 0-377 | 0-415 | 0-307 | 0-150 | 0-208 | | 0-259 |
| **Prevalence** | 79.7% | | 92.0% | 91.9% | 77.8% | 96.8% | 74.7% | 82.4% | 85.3% | | 93.8% |
| Fisher’s test p-value | - | **0.024** | | 0.739 | - | **<0.0001** | 0.739 | - | 0.704 | 0.575 | |
| **Median with zeros** | 29 | | 70 | 72 | 32 | 36 | 89 | 33.5 | 98 | | 76.5 |
| % decreased oocysts load | - | | - | - | - | - | 30.6% | - | - | |  |
| % increased oocysts load | - | | 141.4% | 148.3% | - | 147.2% | - | - | 192.5% | | 128.4% |
| Kruskal-Wallis Comparison Summary | *** | | *** | *** | * | * | * | * | * | | * |
| Dunn’s Multiple Comparison Summary | - | | p<0.05 | p<0.05 | - | ns | ns | - | ns | | ns |
| Mann-Whitney test p-value | - | | **<0.0001** | **0.002** | - | 0.072 | 0.116 | **-** | **0.045** | | **0.036** |
| **Median without zeros** | 39 | | 74.5 | 74 | 87 | 90^^ | 59 | 38.5 | 100 | | 82 |
| Kruskal-Wallis Comparison Summary | ** | | ** | ** | ns | ns | ns | * | * | | * |
| Dunn’s Multiple Comparison Summary | - | | p<0.05 | p<0.05 | - | ns | ns | - | ns | | ns |
| Mann-Whitney test p-value | - | | **0.004** | **0.022** | **-** | 0.795 | 0.070 | **-** | **0.020** | | 0.077 |

^ Removing zeros from this group severely depletes N (from 38 to 15)

^^Removing zeros from this group has little effect on N (from 31 to 30)

*0.05>p>0.03, ** 0.03>p>0.01, *** p>0.01.
