# Supplementary material for: Anopheles Imd Pathway Factors and Effectors in Infection Intensity-Dependent Anti-Plasmodium Action
Source: PLoS Pathog. 2012 Jun 7;8(6):e1002737. doi: 10.1371/journal.ppat.1002737 (PMC3369948; doi:10.1371/journal.ppat.1002737)
Supplement: Table S2 — (A) Early oocysts. (B) Late oocysts. (DOCX) [file ppat.1002737.s002.docx]

**Table S2: (A)** Early oocysts. **(B)** Late oocysts.

**A**.

| **Fig. 3B**  **Early oocysts** | **GFP** | | **Cpr** |
| --- | --- | --- | --- |
| **n** | 68 | | 72 |
| **Range** | 0-143 | | 0-67 |
| **Prevalence** | 95.6% | | 83.3% |
| Fisher’s test p-value | - | **0.005** | |
| **Median with zeros** | 45.5 | | 10.5 |
| % decreased oocysts load | - | | 77.3% |
| Mann-Whitney test p-value | - | | **<0.0001** |

**B.**

| **Fig. 3C**  **Late oocysts** | **GFP** | | **Cpr** |
| --- | --- | --- | --- |
| **n** | 85 | | 85 |
| **Range** | 0-178 | | 0-177 |
| **Prevalence** | 96.5% | | 92.9% |
| Fisher’s test p-value | - | 0.331 | |
| **Median with zeros** | 56 | | 36 |
| % decreased oocysts load | - | | 45.7% |
| Mann-Whitney test p-value | - | | **0.023** |
