# Supplementary material for: Anopheles Imd Pathway Factors and Effectors in Infection Intensity-Dependent Anti-Plasmodium Action
Source: PLoS Pathog. 2012 Jun 7;8(6):e1002737. doi: 10.1371/journal.ppat.1002737 (PMC3369948; doi:10.1371/journal.ppat.1002737)
Supplement: Table S3 — Caspar silencing with and without antibiotics. (DOCX) [file ppat.1002737.s003.docx]

**Table S3:** Caspar silencing with and without antibiotics

| **Fig. 4**  **Oocysts w/ and w/o Ab** | **GFP w/ Ab** | | **GFP w/o Ab** | **Cpr w/ Ab** | **Cpr w/o Ab** |
| --- | --- | --- | --- | --- | --- |
| **n** | 52 | | 51 | 29 | 45 |
| **Range** | 0-190 | | 0-350 | 0-63 | 0-52 |
| **Prevalence** | 80.8% | | 84.3% | 34.5% | 48.9% |
| Fisher’s test p-value | - | 0.572 | | - | 0.060 |
| **Median with zeros** | 36 | | 55 | 0 | 0 |
| % increased oocysts load | - | | 52.8% | - | 0% |
| Mann-Whitney test p-value | - | | 0.072 | - | 0.209 |
| **Median without zeros** | 42 | | 65 | 6.5* | 9.5* |
| Mann-Whitney test p-value | - | | 0.088 | - | 0.729 |

* Removing zeros from these groups severely depletes N (from 29 to 10 and from 45 to 22)
