# Supplementary material for: Anopheles Imd Pathway Factors and Effectors in Infection Intensity-Dependent Anti-Plasmodium Action
Source: PLoS Pathog. 2012 Jun 7;8(6):e1002737. doi: 10.1371/journal.ppat.1002737 (PMC3369948; doi:10.1371/journal.ppat.1002737)
Supplement: Table S4 — (A) Effect of silencing of IMD pathway members and effectors on low exposure infections. (B) Effect of silencing of IMD pathway members and effectors on medium exposure infections. (C) Effect of silencing of IMD pathway members and effectors on high exposure infections. (DOCX) [file ppat.1002737.s004.docx]

**Table S4. (A)** Effect of silencing of IMD pathway members and effectors on low exposure infections. **(B)** Effect of silencing of IMD pathway members and effectors on medium exposure infections. **(C)** Effect of silencing of IMD pathway members and effectors on high exposure infections.

**A.**

| **Fig. 5A**  **Low exposure** | **GFP** | | **WAPL1** | **APL1A** | **APL1B** | **APL1C** | **LRIM** | **TEP1** | **LRRD7** | | **CASPAR** | **IMD** |
| --- | --- | --- | --- | --- | --- | --- | --- | --- | --- | --- | --- | --- |
| **n** | 46 | | 43 | 52 | 48 | 60 | 21 | 46 | 45 | | 49 | 47 |
| **Range** | 0,1-10 | | 0,1-13 | 0,1-11 | 0,1-8 | 0,1-16 | 0,1-12 | 0,1-10 | 0,1-11 | | 0,1-4 | 0,1-9 |
| **Prevalence** | 69.6% | | 86% | 78.8% | 69.4% | 81.7% | 81% | 78.3% | 84.4% | | 51% | 78.7% |
| Fisher’s test p-value | - | **0.0063** | | 0.1933 | 1.0000 | 0.0675 | 0.0718 | 0.1933 | **0.0358** | **0.0090** | | 0.1933 |
| **Median with zeros** | 1 | | 2 | 1 | 2 | 2 | 2 | 2 | 2 | | 1 | 2 |
| % decreased oocysts load | - | | - | - | - | - | - | - | - | | 0% | - |
| % increased oocysts load | - | | 100% | 0% | 100% | 100% | 100% | 100% | 100% | | - | 100% |
| Kruskal-Wallis Comparison Summary | *** | | | | | | | | | | | |
| Dunn’s Multiple Comparison Summary | - | | p>0.05 | p>0.05 | p>0.05 | p>0.05 | p>0.05 | p>0.05 | p>0.05 | | p>0.05 | p>0.05 |
| Mann-Whitney test p-value | - | | **0.0030** | 0.1225 | 0.2433 | **0.0123** | 0.2692 | **0.0253** | **0.0106** | | **0.0124** | 0.1375 |
| **Median without zeros** | 2 | | 3 | 2 | 3 | 3 | 2 | 3 | 2 | | 1 | 2 |
| Kruskal-Wallis Comparison Summary | *** | | | | | | | | | | | |
| Dunn’s Multiple Comparison Summary | - | | p>0.05 | p>0.05 | p>0.05 | p>0.05 | p>0.05 | p>0.05 | p>0.05 | | p>0.05 | p>0.05 |
| Mann-Whitney test p-value | - | | **0.0177** | 0.2442 | **0.0312** | **0.0299** | 0.5896 | **0.0172** | **0.0493** | | 0.0540 | 0.2621 |

*0.05>p>0.03, ** 0.03>p>0.01, *** p>0.01.

**B.**

| **Fig. 5B**  **Medium exposure** | **GFP** | **WAPL1** | **APL1A** | **APL1B** | **APL1C** | **LRIM** | **TEP1** | **LRRD7** | **CASPAR** | | **IMD** |
| --- | --- | --- | --- | --- | --- | --- | --- | --- | --- | --- | --- |
| **n** | 75 | 34 | 32 | 47 | 32 | 38 | 43 | 47 | 59 | | 70 |
| **Range** | 0,1-70 | 0,1-117 | 0,1-111 | 0,1-75 | 0,1-165 | 0,2-122 | 0,1-103 | 0,1-103 | 0,1-85 | | 0,2-140 |
| **Prevalence** | 93.3% | 97.1% | 81.2% | 97.9% | 96.9% | 86.8% | 93% | 95.7% | 62.7% | | 95.7% |
| Fisher’s test p-value | - | 0.2789 | **0.0152** | 0.2789 | 0.4976 | 0.1462 | 1.0000 | 0.7475 | | **< 0.0001** | 0.7475 |
| **Median with zeros** | 7 | 18 | 10 | 16 | 12 | 21.5 | 22 | 18 | 2 | | 22.5 |
| % decreased oocysts load | - | - | - | - | - | - | - | - | 71.4% | | - |
| % increased oocysts load | - | 157.1% | 42.9% | 128.6% | 71.4% | 207.1% | 214.  3% | 157.1% | - | | 221.4% |
| Kruskal-Wallis Comparison Summary | *** | | | | | | | | | | |
| Dunn’s Multiple Comparison Summary | - | p>0.05 | p>0.05 | p>0.05 | p>0.05 | p>0.05 | p>0.05 | **p<0.05** | p>0.05 | | **p<0.05** |
| Mann-Whitney test p-value | - | **0.0032** | 0.8063 | **0.0309** | 0.2427 | **0.0164** | **0.0026** | **0.0004** | **0.0003** | | **0.0005** |
| **Median without zeros** | 9.5 | 19 | 12.5 | 18 | 12 | 25 | 23 | 21 | 6 | | 23 |
| Kruskal-Wallis Comparison Summary | *** | | | | | | | | | | |
| Dunn’s Multiple Comparison Summary | - | p>0.05 | p>0.05 | p>0.05 | p>0.05 | **p<0.05** | **p<0.05** | **p<0.05** | p>0.05 | | **p<0.05** |
| Mann-Whitney test p-value | - | **0.0044** | 0.3728 | 0.0623 | 0.3416 | **0.0006** | **0.0007** | **0.0003** | 0.4082 | | **0.0004** |

*0.05>p>0.03, ** 0.03>p>0.01, *** p>0.01.

**C.**

| **Fig. 5C**  **High exposure** | **GFP** | | **WAPL1** | **APL1A** | **APL1B** | **APL1C** | **LRIM** | **TEP1** | **LRRD7** | | **CASPAR** | **IMD** |
| --- | --- | --- | --- | --- | --- | --- | --- | --- | --- | --- | --- | --- |
| **n** | 80 | | 65 | 69 | 75 | 49 | 41 | 83 | 74 | | 68 | 53 |
| **Range** | 0-373 | | 0-330 | 0-463 | 0-326 | 4-276 | 16-227 | 1-386 | 2-302 | | 0-204 | 11-208 |
| **Prevalence** | 96.2% | | 92.3% | 94.2% | 97.3% | 100% | 100% | 100% | 100% | | 97.1% | 100% |
| Fisher’s test p-value | - | 0.2789 | | **0.0152** | 0.2789 | 0.4976 | 0.1462 | 1.0000 | 0.7475 | **< 0.0001** | | 0.7475 |
| **Median with zeros** | 86 | | 94 | 75 | 83 | 106 | 95 | 120 | 85.5 | | 60.5 | 100 |
| % decreased oocysts load | - | | - | 12.8% | 3.5% | - | - | - |  | | 29.7% | - |
| % increased oocysts load | - | | 9.3% | - | - | 23.3% | 10.5% | 39.5% | 0 | | - | 16.3% |
| Kruskal-Wallis Comparison Summary | *** | | | | | | | | | | | |
| Dunn’s Multiple Comparison Summary | - | | p>0.05 | p>0.05 | p>0.05 | p>0.05 | p>0.05 | p>0.05 | p>0.05 | | p>0.05 | p>0.05 |
| Mann-Whitney test p-value | - | | 0.4105 | 0.7913 | 0.4717 | 0.4303 | 0.3633 | **0.0225** | 0.5912 | | **0.0026** | 0.2936 |
| **Median without zeros** | 93 | | 96.5 | 81 | 85 | 106 | 95 | 120 | 85.5 | | 63 | 100 |
| Kruskal-Wallis Comparison Summary | *** | | | | | | | | | | | |
| Dunn’s Multiple Comparison Summary | - | | p>0.05 | p>0.05 | p>0.05 | p>0.05 | p>0.05 | p>0.05 | p>0.05 | | p>0.05 | p>0.05 |
| Mann-Whitney test p-value | - | | 0.1793 | 0.9575 | 0.3799 | 0.6560 | 0.5548 | 0.0545 | 0.8890 | | **0.0011** | 0.4802 |

*0.05>p>0.03, ** 0.03>p>0.01, *** p>0.01.
