# Supplementary material for: Anopheles Imd Pathway Factors and Effectors in Infection Intensity-Dependent Anti-Plasmodium Action
Source: PLoS Pathog. 2012 Jun 7;8(6):e1002737. doi: 10.1371/journal.ppat.1002737 (PMC3369948; doi:10.1371/journal.ppat.1002737)
Supplement: Table S5 — Primers used for dsRNA synthesis and for verification of knockdown of target genes. (DOCX) [file ppat.1002737.s005.docx]

**Table S5:** Primers used for dsRNA synthesis and for verification of knockdown of target genes and RNAi silencing efficiencies.

| **Gene Name** | **AGAP#** | **Primer Name** | **Primer sequence** | **First description** | **% silencing** |
| --- | --- | --- | --- | --- | --- |
| wAPL1 |  | T7-wAPL1-F | TAATACGACTCACTATAGG ATATAACACTAAACAACC | Mitri *et al.,* 2009 | 81 |
| wAPL1 |  | T7-wAPL1-R | TAATACGACTCACTATAGG AAAGATCCACGTCAACCA | Mitri *et al.,* 2009 |  |
| APL1A | AGAP007036 | T7-APL1A-F | TAATACGACTCACTATAGG ACTACCACCAGCCGAAAGATG | Mitri *et al.,* 2009 | 76 |
| APL1A |  | T7-APL1A-R | TAATACGACTCACTATAGG ATCTGGTCTTGTATAGTACAATGG | Mitri *et al.,* 2009 |  |
| APL1A |  | APL1A-VF | GTAAACGAGCTGAGGACTGCGGTGCAGC | Mitri *et al.,* 2009 |  |
| APL1A |  | APL1A_V_2R | TCCATCTGGTCCTTGAGCTT | - |  |
| APL1B | AGAP007035 | T7-APL1B-F | TAATACGACTCACTATAGG ACTCGCAAAGCTCAGCAAACAC | Mitri *et al.,* 2009 | 86 |
| APL1B |  | T7-APL1B-R | TAATACGACTCACTATAGGAGTGAGAACAAATAAGTTCAAAGTCC | Mitri *et al.,* 2009 |  |
| APL1B |  | APL1B_V_2F | TGCAGATTCTGTTGAGACAGC | - |  |
| APL1B |  | APL1B_V_2R | AACGACGAATCTTGTGTGTTTG | - |  |
| APL1C | AGAP007033 | T7-APL1C-F | TAATACGACTCACTATAGG AGGCCAAGAAGAACCGCAATCC | Mitri *et al.,* 2009 | 79 |
| APL1C |  | T7-APL1C-R | TAATACGACTCACTATAGG ATCACAGTGATTTCAGGGTGTGC | Mitri *et al.,* 2009 |  |
| APL1C |  | APL1C-VF | CTGCTGCAGGGGCTACACGCC | Mitri *et al.,* 2009 |  |
| APL1C |  | APL1C-VR | GGCCCAAGTAACATCATACAC | Mitri *et al.,* 2009 |  |
| LRIM | AGAP006348 | LRIM-RNAi-F | TAATACGACTCACTATAGGCCAAGGCTCGGAAACGGAGCGG | - | 62 |
| LRIM |  | LRIM-RNAi-R | TAATACGACTCACTATAGGTACATATCCCAATCGCGGATGGC | - |  |
| LRIM |  | LRIM-RNAi-VeriF | TACGACGTTAAGGGACAGG | - |  |
| TEP1 | AGAP010815 | Tep1-RNAi-F | TAATACGACTCACTATAGGTTTGTGGGCCTTAAAGCGCTG | Dong et al., 2006 | 92 |
| TEP1 |  | Tep1-RNAi-F | TAATACGACTCACTATAGGACCACGTAACCGCTCGGTAAG | Dong et al., 2006 |  |
| TEP1 |  | Tep1-RNAi-VeriF | GGTGAATCAACGGTACGTTA | Dong et al., 2006 |  |
| LRRD7 | AGAP005693 | LRRD7-RNAi-F | TAATACGACTCACTATAGTCGGTGAGCAACAGTTTGAC | Dong et al., 2006 | 91 |
| LRRD7 |  | LRRD7-RNAi-R | TAATACGACTCACTATAGCTTCATTCCCGCTAATGCTC | Dong et al., 2006 |  |
| LRRD7 |  | LRRD7-RNAi-VeriF | CGCCACGATCGAAAGCACCGCGT | Dong et al., 2006 |  |
| CASPAR | AGAP006473 | CasparRNAi_L | TAATACGACTCACTATAGCCGCTTTTCTAAACGCTGTC | Garver et al., 2009 | 64 |
| CASPAR |  | CasparRNAi_R | TAATACGACTCACTATAGAAACAGGTTGCATGTGTGGA | Garver et al., 2009 |  |
| CASPAR |  | CasparVerify R | GAACGGCTGCGCTTTAACA | Garver et al., 2009 |  |
| IMD | AGAP004959 | IMD-RNAi-F | TAATACGACTCACTATAGGGGAATTTCCCAAATGGTGTG | - | 55 |
| IMD |  | IMD-RNAi-R | TAATACGACTCACTATAGGG TGTGTAGATTGCTCGCGTTC | - |  |
| IMD |  | IMD-Veri-F | CGAGACTATGGCTCACACCA | - |  |
| CaspL1 | AGAP011693 | CaspL1RNAi_L | TAATACGACTCACTATAGGGCGGCGTGGAGAGTAATGTTT |  | 76 |
| CaspL1 |  | CaspL1RNAi_R | TAATACGACTCACTATAGGGTTCGAGCGATGACGTTACTG |  |  |
| CaspL1 |  | CaspL1Verify R | GAAAATGCGCAAAAATTGGT |  |  |
| Rel2 (all) | AGAP006747 | Rel2 rhdRNAi_L | TAATACGACTCACTATAGGGCGGAGAAGTCGAAGAAAACG | Meister, et al., 2005 | 55 |
| Rel2 (all) |  | Rel2 rhdRNAi_R | TAATACGACTCACTATAGGGCACAGGCACACCTGATTGAG | Meister, et al., 2005 |  |
| Rel2-long | AGAP006747 | Rel2 ankRNAi_L | TAATACGACTCACTATAGGGAATCCGACGCAACGATACG | Meister, et al., 2005 | 62 |
| Rel2-long |  | Rel2 ankRNAi_R | TAATACGACTCACTATAGGGGACCGCAATGTGAAGGATG | Meister, et al., 2005 |  |
| Ikk-gamma | AGAP005933 | IkkgRNAi_L | TAATACGACTCACTATAGGGTCTGTCCAAGCACATCGAAC |  | 72 |
| Ikk-gamma |  | IkkgRNAi_R | TAATACGACTCACTATAGGGCACTTGTTCCGCTGTTTTCA |  |  |
| Ikk-gamma |  | IkkgVerify R | AGCCTGCTGTACCATTTTCG |  |  |
| FADD | AGAP007173 | FADDRNAi_L | TAATACGACTCACTATAGGGCTGGCACTGGACACAAAAGA |  | 82 |
| FADD |  | FADDRNAi_R | TAATACGACTCACTATAGGGTTCCAGCTTTTGCCAATTTC |  |  |
| FADD |  | FADDVerify R | TAATACGACTCACTATAGGGCAGCGTCATCGTACTGCATC |  |  |
| TAK1 |  | TAK1RNAi_L | TAATACGACTCACTATAGGGCCGTCGGGAAAGGTTCC |  | 99 |
| TAK1 |  | TAK1RNAi_R | TAATACGACTCACTATAGGGGACGAACCCTCAAACACTTCC |  |  |
| TAK1 |  | TAK1veri | CCGTCGGGAAAGGTTCC |  |  |

F - forward primers, R - reverse primers, and V or Veri –- verification primers. For validation of wAPL1, we used the verification primers for all APL1s (A, B, and C), since wAPL1 (whole APL1) is a fragment of the DNA that is conserved among the three APL1 genes. % silencing: mean knock-down efficiencies from at least three replicates.
